# Beyond expansion: workforce absence, administration and the persistence of NHS elective backlogs

**DOI:** 10.1177/01410768261442040

**Published:** 2026-04-21

**Authors:** Cristina Tealdi, Joan E. Madia, Ahmar Shah, Aziz Sheikh, Catia Nicodemo

**Affiliations:** 1 Department of Economics, Heriot-Watt University, Edinburgh, UK; 2 Department of Primary Care Health Sciences, Medical School, University of Oxford, Oxford, UK; 3 University of Edinburgh, Usher Institute, Edinburgh, UK; 4 Brunel University of London, Middlesex, UK

**Keywords:** NHS productivity, waiting time, elective admission, GMM methods, NHS workforce

## Abstract

**Objective:**

To identify and quantify key factors driving the decline in efficiency of NHS elective care, focusing on medical workforce dynamics, resource allocation and systemic inefficiencies. We hypothesised that medical workforce sickness absence and administrative turnover significantly affect productivity and backlog growth, instead medical workforce turnover has not effect.

**Design:**

This research is a national retrospective observational study using monthly panel data from NHS Digital (January 2018–December 2023). Ordinary Least Squares regression and Generalised Method of Moments models were applied to estimate the impact of workforce and resource factors on productivity and backlog indicators. These methods will account for the unobserved factors and potential reverse causality problem.

**Setting:**

The study follows secondary care across all NHS Trusts in England, with performance measured at the Trust level.

**Participants:**

All NHS Trusts delivering elective surgical services between January 2018 and December 2023. The unit of analysis was Trust-month observations, encompassing all elective surgical patients treated in each Trust.

**Main outcome measures:**

(1) Average per capita completed surgery elective cases (proxy for productivity). (2) Ratio of incomplete elective surgery to average completed elective surgery cases (proxy for additional resources needed to meet demand).

**Results:**

A one-percentage-point increase in NHS medical workforce sickness rates was associated with a 4.4% decrease in average completed elective cases (95% CI −0.0598 to −0.0272, *p* < 0.05). Gains in administrative staff reduced excess elective surgery incomplete cases by 14.4% (95% CI −0.155 to −0.133, *p* < 0.05). Findings were robust to controls for other workforce and resource-related variables.

**Conclusions:**

Workforce expansion alone will not resolve NHS elective surgery backlogs. Reducing medical sickness absence, enhancing staff wellbeing and ensuring adequate administrative capacity are critical to improving productivity and reducing waiting times.

## Introduction

The number of people on NHS England’s waiting list for elective surgery has increased almost threefold since 2013, reaching 7.7 million by early 2024, with only a slight reduction in 2025. Elective care efficiency has fallen markedly: despite workforce expansion, the average number of completed elective procedures per full-time staff member declined by about 4% between 2018 and 2023, and elective waiting lists nearly tripled, from 2.6 million in 2013 to 7.7 million by early 2024.^1,2^ Many patients continue to experience prolonged pain, disability and uncertainty despite an increase in NHS workforce numbers.^3^ The COVID-19 pandemic exacerbated these pressures, causing a 23% decline in productivity through a combination of expanded critical care inputs and widespread cancellation of non-urgent procedures.^4,5^ Persistent underinvestment in infrastructure and limited spare capacity have left the NHS vulnerable to shocks, while financial pressures have constrained service delivery and increased overall costs.^6–8^ In this manuscript, we define NHS productivity as the average number of completed elective surgery cases per clinical workforce member at the Trust level, reflecting how efficiently available staff capacity is translated into delivered care. This measure captures operational efficiency while accounting for differences in workforce size across Trusts.

Workforce challenges remain a critical driver of NHS inefficiencies. High turnover in leadership, the influx of new staff, elevated sickness absence and staff burnout have collectively reduced capacity, engagement and morale.^1,9,10^ High turnover in leadership, the influx of new staff, elevated sickness absence and staff burnout have collectively reduced capacity, engagement and morale. For instance, NHS Digital data show that sickness absence rates rose from 4.2% in 2018 to 5% in 2023, contributing to an estimated 3%–5% decline in elective care productivity across Trusts.^3,4^ The Health Foundation similarly reported a 23% fall in overall NHS productivity during the COVID-19 period, while the 2023 NHS Staff Survey found that one in three employees reported feeling burned out most of the time.^10^

Although recruitment has increased headcount, the system struggles with training pressures and knowledge transfer, while industrial action has further disrupted patient flow.^11^ In addition, infrastructure backlogs and outdated technology impede operational efficiency, and rising costs continue to create trade-offs in resource allocation.^12,13,8^ Despite these challenges, there is limited empirical evidence quantifying the relative contributions of workforce, infrastructure and financial constraints to NHS productivity and waiting times.

We aim to provide a comprehensive analysis of the factors driving elective surgery productivity in NHS England. Using detailed longitudinal data, we quantify the impact of workforce dynamics, resource allocation and systemic inefficiencies on completed and elective surgery incomplete cases. By disentangling the relative importance of staffing shortages, absenteeism, infrastructure deficits and financial pressures, our study provides evidence to guide policy interventions targeted at reducing waiting lists and improving operational resilience.

## Methods

### Overview

We conducted a national retrospective observational study using Trust-level administrative data from NHS England between January 2018 and December 2023. This period was selected because consistent and complete reporting for all key variables, such as elective activity, medical workforce sickness absence and medical and administrative turnover, became available from 2018 onwards. At the time of analysis, December 2023 represented the most recent month for which all relevant datasets (NHS Digital, NHS England and ONS) were publicly released and harmonised across Trusts. Data for early 2024 were still provisional and incomplete for several indicators and therefore were excluded to ensure comparability and reliability across the study period. The study examined trends in elective surgery activity and workforce capacity, focusing on completed elective procedures and incomplete pathways (patients still awaiting elective treatment). We linked these measures to indicators of workforce dynamics (such as medical sickness absence and strike activity), infrastructure investment, demographic factors and neighbouring Trust pressures. The unit of analysis is the NHS Trust observed at the monthly level from January 2018 to December 2023, forming a Trust-month panel dataset. This structure results in repeated monthly observations for each Trust over the study period. Supplemental material contains further details on data sources and modelling strategies.

### Definitions and data sources

These variables were selected based on existing literature, policy relevance and plausibility as contributors to observed variation in elective care delivery. All datasets were drawn from official and publicly accessible sources, including NHS Digital (https://digital.nhs.uk/data-and-information), NHS England (https://www.england.nhs.uk/statistics/) and the Office for National Statistics (ONS; https://www.ons.gov.uk/). Where available, we used data files identified by persistent digital object identifiers (DOIs) or archived dataset reference codes to ensure reproducibility; full dataset metadata and access details are provided in Supplemental Table A1. The analysis period spanned from January 2018 to December 2023, and we used the most recent available data for all datasets included in the analysis.^14,15,16^ For some variables, specifically infrastructure investment and elective surgery incomplete cases within Trusts from 2017 were also used to calculate lag effects, ensuring that temporal dynamics and delayed responses to resource allocation were accurately captured.

### Outcomes definition

Our primary outcome was the monthly number of completed elective surgery pathways per full-time equivalent (FTE) clinical medical workforce member at the Trust level (*Average elective surgery completed cases*). This measure was calculated as the ratio of total completed elective surgery cases in a given Trust-month to the total clinical medical workforce (FTE) in that same month; the term ‘average’ refers to this per-capita ratio rather than an average over time. This measure includes hospital-based doctors and related clinical medical professionals, but excludes nurses, allied health professionals and non-clinical staff. We interpret this variable as a proxy for clinical medical capacity rather than a comprehensive measure of total multidisciplinary surgical system capacity. For all analyses, the denominator includes only clinical medical staff (e.g. doctors and hospital-based clinical professionals) directly involved in patient care, rather than the total NHS workforce. This ensures that the productivity measure reflects the clinical capacity relevant to elective surgery and avoids distortion from non-clinical staff numbers. We used this measure as a proxy for NHS elective surgery care performance, capturing how effectively Trusts were able to deliver planned treatments relative to their staffing capacity.^17,18^ To estimate the additional workforce required to remove hospital backlogs, we analysed the ratio of incomplete elective surgery cases to average elective surgery complete cases at each Trust (*Total incomplete over average complete*). Throughout the manuscript, the term ‘Total elective surgery incomplete cases’ refers specifically to patients on the elective surgery Referral-to-Treatment (RTT) waiting list who had not yet received their planned procedure. This measure (Total incomplete over average complete), therefore, captures the scale of unmet elective surgical demand relative to each Trust’s clinical capacity.

### Variables definition

We included the following control variables in our analysis to account for differences in workforce availability, structural capacity, service demand and organisational stability. *NHS Medical Workforce Sickness Rate* measures short-term medical staff availability and potential medical workforce strain. *NHS Medical Workforce days of strikes* captured periods of industrial action that may have disrupted medical service delivery. *Total Trust Investments in infrastructure (log, one-year lag)* reflected longer-term infrastructure capacity improvements, 1-year lagged to account for delayed effects. To account for service pressure, we included *Total elective surgery Incomplete Cases within Trust (log, one-month lag)* as a measure of internal backlog and *Total elective surgery Incomplete Cases of Trusts within 50* *km (log)* as a proxy for regional system congestion. *Share of People Aged 70+ in Trust local area* served as a demographic control for underlying healthcare demand. *NHS Admin Medical Workforce Turnover (joiners-leavers) and NHS Admin Workforce Turnover (joiners-leavers)* were used to assess organisational stability and continuity, although these variables were only available for a subset of months (from August 2018 to February 2023) and used in robustness checks. Finally, we included *Total Trust Healthcare Resource Groups (HRG) Costs* to adjust for differences in patient case mix and resource intensity across Trusts. Summary statistics are reported in Table 1. Further details around variable definitions are reported in Supplemental Section 1.2.

**Table 1.** Summary statistics.

Variable	January 2018 to December 2018				
	Count	Mean	Std. Dev.	Min	Max
Average elective surgery complete cases (log)	1987	4.076	1.055	−1.991	8.864
Total incomplete over average complete (log)	1987	5.257	1.818	−4.571	8.113
Total elective surgery incomplete cases (log)	1987	9.334	1.789	0	11.575
NHS medical workforce sickness rate	1953	4.281	0.892	2.162	8.124
NHS medical workforce days of strikes	1987	0	0	0	0
Total trust investments in infrastructure (log)	1987	−2.059	1.477	−6.546	4.391
Total elective surgery incomplete cases of trusts within 50 km (log)	1987	9.858	1.422	0.000	10.751
Share of older people (70+) in Trust local area	1708	13.151	2.759	6.739	18.511
Variable	Jan 2023 to Dec 2023				
	Count	Mean	Std. Dev.	Min	Max
Average elective surgery complete cases (log)	2017	3.935	1.13	−4.700	9.643
Total elective surgery incomplete over average complete (log)	2017	6.125	1.893	−2.351	9.536
Total elective surgery incomplete cases (log)	2017	10.060	1.719	3.401	12.376
NHS medical workforce sickness rate	2016	5.013	0.917	2.491	9.004
NHS medical workforce days of strikes	2017	0.584	0.493	0.000	1.000
Total trust investments in infrastructure (log)	2017	−0.799	1.345	−4.826	5.823
Total elective surgery incomplete cases of Trusts within 50 km (log)	2017	10.533	1.489	0.000	11.566
Share of older people (70+) in Trust local area	1742	14.025	3.070	6.823	20.059

### Data cleaning and adjustments

All datasets were harmonised by aligning them to a common monthly time frame (January 2018–December 2023) and matching observations using NHS Trust codes. We applied the NHS Digital Trust-code concordance to resolve Trust mergers and name changes, converted all workforce data to clinical FTE units and ensured that definitions were consistent across sources (e.g. ‘incomplete cases’ referring only to elective RTT cases). These steps produced a unified, comparable Trust-month panel for analysis. In total, the panel included 132 NHS Foundation Trusts out of the 154 active in 2018. We excluded Trusts with incomplete reporting to ensure data consistency across variables and time. This integrated dataset allowed for a detailed investigation into how staffing, infrastructure, population needs and regional pressures affect the performance of elective surgery care across the NHS. For details on data cleaning, variable construction and sources, see Supplemental Section 1.3.

### Statistical analysis

We estimated Ordinary Least Squares (OLS) models to analyse Trust-level performance metrics, with two primary dependent variables: (1) the average elective surgery number of complete cases; and (2) the ratio of elective surgery incomplete cases to average elective surgery complete cases. All specifications include Trust fixed effects, year fixed effects and month fixed effects. Month fixed effects control for seasonality and common monthly shocks affecting all Trusts (including pandemic-related disruptions), while year fixed effects capture broader annual trends. Trust fixed-effect controlled for time-invariant characteristics specific to each NHS Trust. This comprehensive approach ensured that our estimates were robust to a wide range of potential confounding factors. For many of the variables in our analysis, we applied logarithmic transformations to improve interpretability, stabilise variance or linearise associations in the data (see Supplemental Material for details). When the dependent variable is log-transformed and explanatory variables are included in levels (e.g. sickness rate measured in percentage points), coefficients are interpreted as semi-elasticities.

Three progressively adjusted models were specified: Model 1 (M1) included baseline fixed effects; Model 2 (M2) added the 1-month lag (*t*−1) of elective surgery incomplete cases at the Trust level, reflecting short-term persistence in backlog dynamics; and Model 3 (M3) further incorporated the local population share of adults aged 70 years or older. Standard errors were reported alongside coefficient estimates, with statistical significance assessed using *t*-tests, exact *p*-values and confidence intervals (CIs). Model fit was evaluated using the *R*^2^- statistic, which quantified the proportion of variance in the outcome explained by the predictors. To maximise statistical power and minimise potential bias, we retained all available observations in the analysis rather than employing listwise deletion of cases with missing data. This analytical approach robustly estimates the associations while controlling for both observed confounders and unobserved heterogeneity across time and organisational units.

To complement our OLS results and address potential econometric concerns, we employed a System Generalised Method of Moments (GMM) estimator. This approach offers two key advantages for panel data: it captures dynamic relationships (where current outcomes depend on past values) and addresses endogeneity, particularly reverse causality. We instrumented the lagged dependent variable (elective surgery complete cases at *t*−1, i.e, 1 month lag) using deeper lags to break potential feedback loops, satisfying the exclusion restriction since the lags affect current outcomes only indirectly via past outcomes.

The GMM framework also accounts for serial correlation via lagged terms, omitted variable bias through fixed effect (Trust, year and month) and endogeneity via internal instrumentation. It accommodates operational inertia common in healthcare systems, while assuming that current outcomes do not influence past inputs, a plausible assumption given the budgetary allocation cycles in the NHS. Our specification retains the same fixed-effect as the OLS models, ensuring comparability and is applied across all three model specifications (M1–M3).

Supplemental Section 2 provides more details on these modelling strategies. We used Stata 18 for the analysis.

## Results

**Figure 1.**
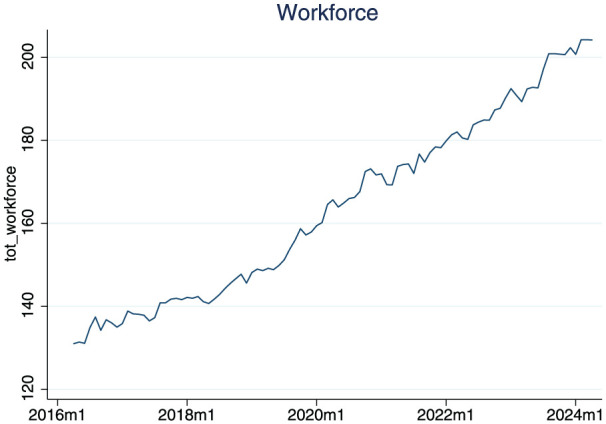
Total clinical medical workforce (TFE) and total number of complete and incomplete cases in England.

Figure 1, Panel A, shows the average total NHS workforce at the Trust level from January 2018 to December 2023. Workforce numbers grew steadily over time, reflecting broader system expansions. Panel B displays the trends in elective surgery completed and incomplete cases. Completed cases remained relatively stable, except for a notable drop during the COVID-19 pandemic, while incomplete cases were already rising pre-pandemic and surged further post-2020, from around 5 to over 9 million monthly.

Table 1 provides summary statistics for key variables across 2018 and 2023. Between these years, the average number of elective surgery complete cases (log) declined from 4.07 to 3.94, while elective surgery incomplete cases (log) rose from 9.26 to 10.06. The ratio of elective surgery incomplete to average elective surgery complete cases (log) also increased, from 5.19 to 6.13. The NHS sickness rate rose from 4.28% to 5.01%, and strike activity emerged in 2023 (mean = 0.58 strike months per Trust). Infrastructure investment (log) increased from −2.12 to −0.80, though variation across Trusts remained substantial. Additionally, elective surgery incomplete cases in neighbouring Trusts (within 50 km) increased, and the local population aged 70+ rose from 12.90% to 14.03%, highlighting demographic shifts.

Table 2 presents OLS estimates of factors associated with elective surgery complete cases (log). The NHS sickness rate was consistently negative and statistically significant: a one-percentage-point increase was linked to a 3.7% to 4.3% reduction in elective surgery complete cases (*p* < 0.01). Strike days had no significant effect, suggesting minimal impact on routine reporting. In Models M2 and M3, 1-month lagged elective surgery incomplete cases (log) were positively associated with elective surgery complete cases. In M3, a 1% increase in lagged elective surgery incomplete cases was linked to a 0.076% rise in elective surgery complete cases (*p* < 0.01), possibly reflecting corrective behaviours. The share of older adults (70+) had a significant negative effect in M3: a one-percentage point increase corresponded to a 13.1% decline in complete case reporting (*p* < 0.01), likely due to added administrative demands. Infrastructure investment remained statistically insignificant, while elective surgery incomplete cases in neighbouring Trusts had a modest but significant positive effect (coefficient = 0.066, *p* < 0.05), pointing to possible regional peer effects or shared capacity pressures.

**Table 2.** OLS estimates of effects on average elective surgery completed cases (log) at NHS Trust level (2018–2023).

Variables Name	M1	M2	M3
NHS medical workforce sickness rate	−0.0370*** (0.0101) *p* = 0.000262 CI: [−0.0569, −0.0171]	−0.0404*** (0.0101) *p* = 6.67e-05 [−0.0602, −0.0205]	−0.0433*** (0.00795) *p* = 5.28e-08 [−0.0589, −0.0277]
NHS medical workforce days of strikes	−0.0225 (0.0312) *p* = 0.471 CI: [−0.0836, 0.0386]	−0.0281 (0.0311) *p* = 0.367 [−0.0891, 0.0329]	0.000691 (0.0246) *p* = 0.978 [−0.0475, 0.0488]
Total trust investments in infrastructure (log, lag)	0.00140 (0.00753) *p* = 0.852 CI: [−0.0134, 0.0162)	0.00203 (0.00752) *p* = 0.787 [−0.0127, 0.0168]	0.0127* (0.00712) *p* = 0.0747 [−0.00126, 0.0266]
Total elective surgery incomplete cases of trusts within 50 km (log)	0.0702** (0.0351) *p* = 0.0453 CI: [0.00148, 0.139]	0.0673* (0.0350) *p* = 0.0544 [−0.00127, 0.136]	0.0665** (0.0262) *p* = 0.0112 [0.0151, 0.118]
Total elective surgery incomplete cases within trusts (log, lag)		0.189*** (0.0237) 0 CI: [0.142, 0.235]	0.0758*** (0.0203) 0.000196 [0.0359, 0.116]
Share of older people (70+) in trust local area			−0.131*** (0.0437) *p* = 0.00265 CI: [−0.217, −0.0457]
Observations	11,733	11,720	10,173
*R* ^2^	0.526	0.529	0.570
Trust fixed effects	Yes	Yes	Yes
Year fixed effects	Yes	Yes	Yes
Month fixed effects	Yes	Yes	Yes

Standard errors in parentheses. *p*-values prefixed with ‘*p* =’. Confidence intervals in square brackets.

*** *p* < 0.01, **p < 0.05, *p < 0.1.

Table 3 shows OLS models estimating the log ratio of elective surgery incomplete to complete cases. The NHS sickness rate had a consistent positive association across all models. In M1, a one-percentage-point rise in sickness was associated with a 4.7% increase in the ratio (*p* < 0.01), reducing slightly to 3.7% in M3 (*p* < 0.01), even after including lagged variables and demographics. Strike days remained statistically insignificant in all models. Lagged elective surgery incomplete cases (log) had a strong effect: in M3, a 1% point increase led to a 0.82% point increase in the incomplete-to-complete ratio (*p* < 0.01), suggesting persistent reporting challenges. The share of older individuals (70+) had a significant and positive association in M3: a one-percentage-point increase correlated with a 13.4% rise in the incomplete-to-complete ratio (*p* < 0.01), reflecting the administrative complexity in ageing populations. Neighbouring Trusts’ incomplete case volumes became significant only in M3, with a negative coefficient (−0.0675, *p* < 0.05), possibly indicating benchmarking or local spillover effects. Infrastructure investment (1-year lagged) had a significant and negative association in M3: a one-percentage-point increase correlated with a 1.55% decline in the incomplete-to-complete ratio (*p* < 0.05), suggesting that capital inputs may influence reporting performance.

**Table 3.** OLS estimates of elective surgery incomplete over average complete cases (log) at NHS Trust level, 2018 to 2023.

Variables Name	M1	M2	M3
NHS medical workforce sickness rate	0.0473*** (0.0107) *p* = 9.67e-06 CI: [0.0264, 0.0683]	0.0326*** (0.0103) *p* = 0.00163 [0.0123, 0.0528]	0.0368*** (0.00821) *p* = 7.52e-06 [0.0207, 0.0529]
NHS medical workforce days of strikes	0.0465 (0.0329) *p* = 0.158 CI: [−0.0180, 0.111]	0.0331 (0.0318) *p* = 0.298 [−0.0292, 0.0953]	0.00181 (0.0254) *p* = 0.943 [−0.0479, 0.0516]
Total trust investments in infrastructure (log, lag)	−0.0113 (0.00795) *p* = 0.156 CI: [−0.0269, 0.00431]	−0.00473 (0.00768) *p* = 0.538 [−0.0198, 0.0103]	−0.0155** (0.00736) *p* = 0.0354 [−0.0299, −0.00106]
Total elective surgery Incomplete cases of Trusts within 50 km (log)	−0.0557 (0.0370) *p* = 0.133 CI: [−0.128, 0.0169]	−0.0666* (0.0357) *p* = 0.0620 [−0.137, 0.00334]	−0.0675** (0.0271) *p* = 0.0127 [−0.121, −0.0144]
Total elective surgery Incomplete cases within Trust (log, lag)		0.709*** (0.0242) *p* = 0 CI: [0.662, 0.756]	0.823*** (0.0210) *p* = 0 [0.782, 0.864]
Share of older people (70+) in Trust local area			0.134*** (0.0452) *p* = 0.00301 CI: [0.0455, 0.223]
Observations	11,733	11,720	10,173
*R* ^2^	0.839	0.850	0.888
Trust fixed effects	Yes	Yes	Yes
Year fixed effects	Yes	Yes	Yes
Month fixed effects	Yes	Yes	Yes

*Notes:* Standard errors in parentheses. *p*-values prefixed with ‘*p* =’. Confidence intervals in square brackets.

*** *p* < 0.01, **p < 0.05, *p < 0.1.

### Robustness checks

Table 4 extends the prior analysis in Tables 2 and 3 by incorporating three additional variables – NHS Medical Workforce Turnover, NHS Administrative Workforce Turnover and HDGR cost – while estimating their effects on both per capita elective surgery complete cases and incomplete cases over average at the NHS Trust level. However, these data are only available for a subset of months (from August 2018 to February 2023), meaning that we lose approximately 1 year of observations. Despite having a year less, the results remained broadly consistent with previous findings, while also highlighting new insights regarding organisational dynamics and financial factors. As in earlier models, the NHS medical sickness rate continued to exhibit a strong and statistically significant association with the completeness of elective surgery case reporting. Specifically, it was negatively associated with per capita elective surgery complete cases (coefficients ranging from −0.0432 to −0.0559, *p* < 0.01) and positively associated with the ratio of elective surgery incomplete cases over average (*p* < 0.01). These findings underscore the critical impact of workforce health on both the quantity and quality of case reporting. The NHS Medical Workforce Turnover variable was consistently non-significant across all models. The NHS Admin Workforce Turnover variable was instead consistently significant across all models. It has a positive impact on per elective surgery capita complete cases (0.0156 in M2, *p* < 0.01) and a negative impact on incomplete ratios (−0.0173 in M2, *p* < 0.01), indicating that administrative stability positively affects data completeness and efficiency. Higher NHS administrative workforce turnover can disrupt scheduling, data processing and coordination functions that underpin patient flow, meaning that greater instability in administrative teams can ultimately slow operational processes and contribute to longer waiting times. As in Tables 2 and 3, 1-month lagged elective surgery incomplete cases within Trusts remained a highly significant and positive predictor of incomplete ratios, reinforcing the persistence of internal reporting challenges. Similarly, total incomplete cases within 50 km maintain their relevance, with positive and significant coefficients in both sets of models, supporting the view that regional dynamics continue to shape Trust-level performance. Interestingly, infrastructure investment exhibits a positive and significant association with per capita elective surgery complete cases in M2 (*p* < 0.05), but a negative and significant association with incomplete ratios in M2 (*p* < 0.01), suggesting that capital investment may aid performance. The share of older adults (70+) remains statistically significant in all specifications, suggesting demographic pressures are strong predictors of reporting outcomes within this sample. HDGR cost, introduced as a proxy for patient complexity and workload, does not emerge as a significant predictor of the incomplete ratio model. Overall, these results highlight the critical roles of administrative stability, infrastructure and local competitive pressures in shaping data completeness, while underscoring the need for richer and more uniform data coverage to further refine NHS performance insights.

**Table 4.** OLS estimate effects of average elective surgery complete cases (log) and incomplete cases over average complete cases (log) at NHS Trust level, August 2018 to February 2023.

	Per capita		Total incomplete over	
Variables Names	Complete		Average complete	
NHS medical workforce sickness rate	−0.0432*** (0.00835) *p* = 2.34e-07 CI: [−0.0595, −0.0268]	−0.0559*** (0.00855) *p* = 6.86e-11 [−0.0726, −0.0391]	0.0364*** (0.00860) *p* = 2.30e-05 [0.0196, 0.0533]	0.0469*** (0.00883) *p* = 1.12e-07 [0.0296, 0.0643]
NHS workforce days of strikes	−0.0607 (0.0524) *p* = 0.247 CI: [−0.163, 0.0420]	−0.177** (0.0721) *p* = 0.0143 [−0.318, −0.0353]	0.0482 (0.0540) *p* = 0.372 [−0.0576, 0.154]	0.165** (0.0746) *p* = 0.0267 [0.0191, 0.311]
Total trust investments in infrastructure (log, lag)	0.0138* (0.00818) *p* = 0.0918 CI: [−0.00224, 0.0298]	0.0308*** (0.00953) *p* = 0.00125 [0.0121, 0.0494]	−0.0181** (0.00843) *p* = 0.0314 [−0.0347, −0.00162]	−0.0366*** (0.00985) *p* = 0.000204 [−0.0559, −0.0173]
Total elective surgery Incomplete cases of Trusts within 50 km (log)	0.0966*** (0.0297) *p* = 0.00114 CI: [0.0384, 0.155]	0.0985*** (0.0286) *p* = 0.000588 [0.0424, 0.155]	−0.0984*** (0.0306) *p* = 0.00129 [−0.158, −0.0385]	−0.103*** (0.0296) *p* = 0.000489 [−0.161, −0.0453]
Total elective surgery incomplete cases within Trust (log, lag)	0.0782*** (0.0239) *p* = 0.00108 CI: [0.0313, 0.125]	0.115*** (0.0276) *p* = 3.32e-05 [0.0606, 0.169]	0.822*** (0.0246) *p* = 0 [0.773, 0.870]	0.779*** (0.0286) *p* = 0 [0.723, 0.835]
Share of old people (70+) in Trust local area	−0.183*** (0.0648) *p* = 0.00483 CI: [−0.310, −0.0556]	−0.256*** (0.0818) *p* = 0.00178 [−0.416, −0.0953]	0.177*** (0.0667) *p* = 0.00786 [0.0466, 0.308]	0.254*** (0.0846) *p* = 0.00271 [0.0879, 0.419]
NHS medical workforce turnover (joiners–leavers)	−0.00181 (0.00132) *p* = 0.171 CI: [−0.00441, 0.000785]	−0.000735 (0.00187) *p* = 0.695 [−0.00441, 0.00294]	0.00147 (0.00136) *p* = 0.280 [−0.00120, 0.00415]	0.00140 (0.00194) *p* = 0.471 [−0.00240, 0.00519]
NHS admin workforce turnover (joiners–leavers)	0.00716*** (0.000462) *p* = 0 CI: [0.00626, 0.00807]	0.0156*** (0.000742) *p* = 0 [0.0141, 0.0170]	−0.00764*** (0.000476) *p* = 0 [−0.00857, −0.00670]	−0.0173*** (0.000767) *p* = 0 [−0.0188, −0.0158]
Total HGR cost		−0.0768 (0.0695) *p* = 0.269 CI: [−0.213, 0.0594]		0.120* (0.0718) *p* = 0.0954 [−0.0210, − 0.260]
Observations	7773	5504	7773	5504
*R* ^2^	0.600	0.661	0.892	0.903
Trust fixed effects	Yes	Yes	Yes	Yes
Year fixed effects	Yes	Yes	Yes	Yes
Month fixed effects	Yes	Yes	Yes	Yes

*Notes:* Standard errors in parentheses. *p*-values prefixed with ‘*p* =’. Confidence intervals in square brackets.

*** *p* < 0.01, **p < 0.05, *p < 0.1.

To assess the robustness of our findings, we re-estimated the main specifications using GMM to address potential endogeneity. The GMM results reinforced the core conclusions from the OLS models, particularly highlighting the persistent and statistically significant impact of NHS staff sickness rates and regional incomplete case pressures on data completeness. Additionally, administrative turnover emerges as a strong predictor of both complete and incomplete case ratios, underscoring the importance of organisational stability. These findings remained broadly consistent across multiple model specifications, present a higher value than OLS to address some issues of endogeneity. Full regression outputs and extended discussion are provided in Supplemental Section 3, Tables A2 to A4.

## Discussion

Our analysis highlights the complex interplay between workforce dynamics, resource allocation and systemic inefficiencies in the NHS, particularly in the context of managing elective surgery waiting lists. Despite an increase in the NHS workforce of approximately 250,000 FTE between 2017 and 2023, the backlog of incomplete elective surgery cases has continued to grow, reaching an alarming 7.7 million cases in early 2024, representing approximately 11.5% of the total population. One of the most significant findings was the strong positive association between staff sickness rates and the ratio of elective surgery incomplete to complete cases. Higher medical sickness rates were consistently associated with a decrease in elective surgery complete cases across all models (see Table 2). This underscores the impact of staff burnout and persistent sick leave on healthcare productivity, particularly in the post-pandemic era. The lingering effects of COVID-19, combined with pre-existing challenges, have left the NHS with a workforce that is overstretched and under significant strain. Addressing staff burnout and improving workforce resilience must therefore be a priority for policymakers seeking to reduce the backlog of elective surgeries.

The findings from our robustness checks using the GMM model further supported the validity of our results. The GMM analysis has confirmed the significant impact of staff sickness rates and diagnostic activity on elective surgery incomplete cases, while also highlighting the importance of controlling for dynamic associations and endogeneity in healthcare service delivery (Supplemental Tables A2–A4). Another critical factor was administrative turnover, which has shown a significant negative association with elective surgery incomplete cases (Table 4). This suggests that higher turnover rates, particularly among experienced staff, can disrupt institutional knowledge transfer and training systems, further exacerbating inefficiencies in healthcare delivery. The recovery phase following the pandemic has been marked by substantial turnover in leadership and management roles, alongside an influx of new staff to address workforce shortages.^10^ While this has increased overall headcount, it has placed additional pressure on the system, highlighting the need for targeted interventions to stabilise the workforce and improve retention. These results suggest that reducing persistent sick leave, investing in infrastructure and coordinating regionally are robust strategies for improving NHS Trust performance. On the other hand, increased investments in healthcare infrastructure were associated with an increase of elective surgery complete cases (Table 4). This highlights the importance of long-term investments in improving system capacity and resilience. However, the variability in healthcare delivery across different Trusts suggests that investments must be targeted to address disparities in access and outcomes.^7^

Taken together, these findings highlight the importance of internal capacity and demographic pressures in shaping data quality across NHS Trusts. They suggest that targeted efforts to reduce staff sickness and address persistent underperformance, particularly in Trusts serving older populations, may be more effective in improving reporting completeness than broader infrastructure investments.

Our findings align with reports by the NHS and UK Government aimed at understanding why NHS productivity has not improved despite increased workforce and investment. For instance,^19^ concluded that, although funding and staffing have risen since the height of the COVID-19 pandemic, the NHS in England is experiencing a productivity crisis due to low capital investment, high staff turnover, under-management and unclear incentives, leading to reduced activity, long waiting times and poorer outcomes.^19^ Along the same line,^20^ found that a decline in employees’ voluntary contributions beyond their core duties could be one factor behind the observed decrease in healthcare system efficiency following the COVID-19 pandemic.

Despite revealing these insights, our study has several limitations that constrain the strength of the conclusions. Firstly, the reliance on Trust-level administrative data provides a broad but coarse view of NHS operations and cannot fully capture underlying clinical processes, case-mix variation or detailed workforce dynamics. As a result, the associations we report should not be interpreted causally. Secondly, our analysis focuses specifically on elective surgery waiting lists, meaning that the findings may not generalise to urgent, emergency or outpatient care. This limits the scope of the policy implications. Thirdly, some datasets, including HRG costs, were unavailable for all Trusts. We therefore use HRG costs only in supplementary checks, and we recognise that they provide an indirect and imperfect proxy for surgical complexity. Although the inclusion of Trust fixed effects helps account for structural differences across Trusts, data limitations may still affect the precision of our estimates. Finally, note that our productivity measure captures clinical medical workforce capacity and does not directly account for the broader multidisciplinary teams (e.g. nursing, anaesthetic and theatre staff) that also contribute to surgical throughput. Overall, these constraints imply that our findings should be viewed as descriptive patterns rather than prescriptive evidence about staffing policy. We explicitly acknowledge that further research using richer clinical or patient-level data is needed before drawing firm conclusions for NHS workforce strategy.

## Conclusion

In conclusion, our study provides evidence-informed insights into the factors driving the rise in incomplete elective surgery cases in the NHS and an improved understanding of the NHS struggles to increase capacity despite the increase in staff numbers. Our findings highlight the need for a multifaceted approach to address workforce challenges, improve resource allocation and enhance system resilience. Policymakers must prioritise interventions that reduce staff burnout, stabilise the workforce and increase investments in healthcare infrastructure to ensure that the NHS can meet the growing demand for services in the post-pandemic era.

## Supplemental Material

sj-pdf-1-jrs-10.1177_01410768261442040 – Supplemental material for Beyond expansion: workforce absence, administration and the persistence of NHS elective backlogs Supplemental material, sj-pdf-1-jrs-10.1177_01410768261442040 for Beyond expansion: workforce absence, administration and the persistence of NHS elective backlogs by Cristina Tealdi, Joan E. Madia, Ahmar Shah, Aziz Sheikh and Catia Nicodemo in Journal of the Royal Society of Medicine

## Declarations

**Ethical approval:** The study relied on publicly available data and thus was considered exempt from review.

**Guarantor:** CN.

**Contributorship:** AS, CN, CT, JM and AS contributed to the design of the study. CN and CT contributed to the data extraction for this work and to the analysis of the data. All authors contributed to the data visualisation and manuscript writing. All authors reviewed and validated this manuscript. The corresponding author attests that all listed authors meet authorship criteria and that no others meeting the criteria have been omitted.

**Provenance:** Not commissioned; peer-reviewed by Julie Morris.
