# Supplementary material for: Beyond expansion: workforce absence, administration and the persistence of NHS elective backlogs
Source: J R Soc Med. 2026 Apr 21;119(5):131–40. doi: 10.1177/01410768261442040 (PMC13099723; doi:10.1177/01410768261442040)
Supplement: sj-pdf-1-jrs-10.1177_01410768261442040 – Supplemental material for Beyond expansion: workforce absence, administration and the persistence of NHS elective backlogs [file sj-pdf-1-jrs-10.1177_01410768261442040.pdf]

Supplementary material

Table of Contents

1 Data **2**

1.1 Data Sources 2

1.2 Variables definition 2

1.3 Data cleaning and adjustments 3

2 Additional Methodological Details **4**

2.1 OLS regression 4

2.2 Extended GMM Specification 5

3 Robustness checks **5**

1 Data

1.1 Data Sources

Table A1: Data sources, frequencies, availability, and identifiers.

Data source	Frequency	Availability	Source / Identifier (URL or DOI)
Referral to Treatment (RTT) Waiting Times	Monthly	2017–2023	NHS England, https://www.england.nhs.uk/statistics/statistical-work-areas/rtt-waiting-times/
NHS Medical Sickness Rates	Monthly	2017–2023	NHS Digital, https://digital.nhs.uk/data-and-information/publications/statistical/nhs-sickness-absence-rates
NHS Medical Workforce days of strikes	Daily	2022–2023	NHS England, https://www.england.nhs.uk/statistics/statistical-work-areas/
NHS Workforce Statistics	Monthly	2017–2024	NHS Digital, https://digital.nhs.uk/data-and-information/publications/statistical/nhs-workforce-statistics
NHS Infrastructure Investments (ERIC)	Yearly	2017–2023	NHS England ERIC, https://www.england.nhs.uk/statistics/statistical-work-areas/estates-returns-information-collection-eric/
ONS Population Estimates (Share 70+)	Yearly	2017–2023	Office for National Statistics, https://www.ons.gov.uk/peoplepopulationandcommunity/populationandmigration/populationestimates
NHS Medical Turnover (Joiners–Leavers)	Monthly	Aug 2018–Feb 2023	NHS Digital, https://digital.nhs.uk/data-and-information/publications/statistical/nhs-workforce-statistics
NHS Admin Turnover (Joiners–Leavers)	Monthly	Aug 2018–Feb 2023	NHS Digital, https://digital.nhs.uk/data-and-information/publications/statistical/nhs-workforce-statistics
Trust HRG Costs / Case-Mix Index	Yearly	2018–2023	NHS England Costing, https://www.england.nhs.uk/costing-in-the-nhs/

1.2 Variables definition

We included several variables as control factors in the analysis. We selected the NHS sickness absence rate as a core measure of workforce availability (*NHS Medical Workforce Sickness rate*). Sickness absence is a critical determinant of service capacity, particularly in settings such as elective surgery care, where delays are sensitive to marginal changes in staffing. High absenteeism rates can reflect workplace stress, poor staff well-being, or outbreaks of infectious diseases, all of which can lead to cancellations or reduced performance. NHS Digital defines absenteeism at work and provides information on whether the absence is due to sickness. This publication presents sickness absence rates for NHS medical staff, based on data from the Electronic Staff Record (ESR).

To capture external disruptions to service delivery, we have used NHS medical workforce strike days to construct a monthly indicator identifying periods of industrial action involving NHS doctors (*NHS Workforce days of strikes*). These events may cause significant short-term reductions in service availability, potentially leading to a temporary backlog increase. We have also included total investments at the trust level to reflect long-term structural capacity (*Total Trust Investments in infrastructure (log, one-year lag)*). Capital investment in infrastructure such as hospital expansions, upgraded diagnostic equipment, and digital health systems is intended to improve throughput and service quality. However, such effects are not immediate. To capture this relationship, we have applied a one-year lag to the investment variable and adjusted for workforce size by constructing a per capita measure. A logarithmic transformation was used to address the right-skewed distribution of investment levels across trusts (see Table A.2). To reflect regional operational pressures, we have included two variables: *Total elective surgery Incomplete cases within Trust (log, lag)* and *Total elective surgery Incomplete cases of Trusts within 50km (log)*. The former serves as a direct measure of internal backlog, capturing the accumulated demand for care at the trust level. The latter aggregates elective surgery incomplete cases across all neighbouring trusts within a 50 km distance from the trust, providing a proxy for local healthcare system congestion. This variable captures competition for shared resources such as specialist staff or diagnostics and potential spillovers from nearby high-demand areas. Both variables have been log-transformed due to their right-skewed distribution (see Table A.2). To account for demographic variation in healthcare needs, we have included the *Share of people aged 70+ in Trust local area*, based on ONS data. Older populations are disproportionately likely to require elective procedures, including orthopaedic, ophthalmologic, and cardiovascular interventions. This variable serves as a key proxy for latent demand, enabling adjustment for underlying variations in population health profiles across trust catchment areas.

We use both the NHS medical staff turnover rate and the NHS administrative staff turnover rate as measures of organisational stability (*NHS Medical Workforce turnover (joiners – leavers)* and *NHS Admin Workforce turnover (joiners – leavers)*. Clinical turnover is particularly important because high rates of medical staff churn can disrupt continuity of care, weaken team-specific human capital, and reduce the capacity of trusts to maintain consistent standards in diagnosis, treatment, and follow-up, with potential implications for patient safety and outcomes. Administrative staff turnover is also a key indicator of institutional stability and can significantly affect operational efficiency and service delivery. High administrative turnover can impair continuity in care coordination, resource planning, and patient scheduling. These metrics, calculated as the net difference between joiners and leavers divided by medical workforce size, capture the extent to which internal churn may undermine both clinical performance and operational efficiency. Their inclusion allows us to test whether more stable trusts perform better, even after adjusting for clinical workforce size. Lastly, we have included Total Trust HRG costs (*Total Trust HRG costs*), an aggregate measure of Healthcare Resource Group expenditures. This variable serves as a proxy for case mix intensity and resource consumption. Trusts facing higher-cost patients or more complex procedures may have limited capacity to accelerate throughput, even with similar staffing levels. Controlling for HRG costs helps isolate whether performance differences reflect efficiency rather than differences in patient complexity. The cost variable has been log-transformed due to its right-skewed distribution (see Table A.2).

1.3 Data cleaning and adjustments

All variables were harmonised and linked using NHS Trust codes. The data cover the period 2018 to 2023 (see Supplementary material Tables A2 and A3 for more details). The two primary outcome variables were derived from monthly Referral to Treatment (RTT) data at the Trust level , available from January 2017 to December 2023. The number of elective surgery completed cases was calculated by aggregating inpatient and non-admitted pathways across all waiting times and treatment categories. Elective surgery incomplete cases were calculated similarly, including those with and without a decision-to-admit (DTA) status, capturing the full scope of treatment backlogs. Workforce data were obtained from NHS Digital, providing monthly estimates of full-time equivalent (FTE) staff across all medical specialities. These data were used to construct the denominators for both outcome measures. The first outcome variable was computed as the ratio of total elective surgery completed cases in a given Trust-month to the total Trust-level clinical medical workforce (FTE) in that same month. The term 'average' refers to this per-capita monthly ratio and does not represent an average across time. The second, elective surgery total incomplete over elective surgery average completed, was calculated as the ratio of elective surgery incomplete cases to the average number of elective surgery completed cases, capturing backlog pressure relative to delivery

capacity. Both variables were then logged to normalise their distributions and reduce the influence of extreme values, facilitating interpretation and comparability in the regression analyses (See Table A.2). To account for variation in staff availability, we included the NHS sickness rate, sourced from monthly datasets published by NHS Digital for the period January 2018 to December 2023. This variable measured the proportion of NHS staff absent due to illness, calculated as sickness absences divided by the total NHS workforce. NHS strike activity was captured using daily data compiled by NHS Wiki Data for the period 2022 to 2023. For earlier periods, the variable was imputed as zero. A binary monthly indicator was constructed, equal to one if at least one strike day occurred within a given month. NHS infrastructure investment was measured using the Estates Returns Information Collection (ERIC), also provided by NHS Digital. Annual investment figures were calculated by summing capital expenditure on new buildings, existing infrastructure improvements, maintenance, and equipment procurement, covering the years 2017 to 2023. The resulting variable was log-transformed to normalise its distribution. Demographic pressure was proxied by the share of the population aged 70 and above, based on annual age- and gender-specific population estimates published by the ONS. As these data were reported at the local authority level, each NHS Trust was matched to the appropriate local authority or group of authorities. To capture organisational stability, we included the medical and administrative turnover rates, calculated using monthly NHS Digital data on joiners and leavers across staff categories from August 2018 to February 2023. We included medical and administrative personnel separately, due to differences in their roles and responsibilities. This separation allows us to capture potentially heterogeneous effects on organisational stability and to avoid conflating clinical capacity with administrative support functions. Turnover was defined as the net difference between joiners and leavers, divided by the total medical workforce. Unfortunately, the information was available only between August 2018 and February 2023. Finally, total HRG costs were included as an indirect indicator of case-mix complexity and healthcare intensity. These data, sourced from NHS Digital, are reported annually and were available for the years 2018 to 2023. The variable was log-transformed to improve comparability across Trusts and to reduce the influence of very large values. As HRG costs were available for only around half of NHS Trusts during the study period (see Table A3), we used this measure solely in supplementary robustness checks rather than in the main models. A chi-squared test does not reject independence between region and missingness of the cost variable (Pearson chi2(6) = 3.8930, p= 0.691). Although HRG costs provide only a broad proxy for case-mix complexity, they represent the only nationally consistent indicator available for all relevant years.

2 Additional Methodological Details

2.1 OLS regression

We employ an ordinary least squares (OLS) regression to examine the relationship between the log of elective surgery average complete cases at the NHS Trust level and a range of explanatory variables. The general form of the estimation equation is specified as:

$$\ln(Y_{imt}) = \beta_1 X_{1,imt} + \beta_2 X_{2,imt} + \ldots + \beta_k X_{k,imt} + \mu_i + \sigma_i + \lambda_t + \epsilon_{imt} \quad (1)$$

where:

- Y_{it} represents the elective surgery average complete cases (log-transformed) for Trust i in month m in the year t,
- $X_{k,it}$ denotes the kth explanatory variable, such as NHS workforce sickness rate, strike days, infrastructure investments, elective surgery incomplete cases within 50km, and the share of older adults in the Trust area,
- μ_i are Trust fixed effects to control for time-invariant unobserved heterogeneity,
- σ_i are month fixed effects to capture the seasonality
- λ_t are year fixed effects to capture time-specific shocks affecting all Trusts,
- ϵ_{it} is the error term.

All continuous variables are either log-transformed or lagged to address potential skewness and capture dynamic relationships. Standard errors are clustered at the Trust level to account for potential within-Trust correlation over time.

We have also estimated using the same model the outcome variable defined as the ratio of elective surgery incomplete cases to elective surgery average completed cases across NHS Trusts ($\frac{\text{Incomplete cases}_{it}}{\text{Average complete cases}_{it}}$).

2.2 Extended GMM Specification

We utilise the Generalised Method of Moments (GMM) approach to estimate the relationship between the ratio of elective surgery incomplete cases to elective surgery average completed cases and a set of explanatory variables. GMM is particularly suited for addressing potential endogeneity concerns, where some explanatory variables may be correlated with the error term ϵ_{it}. Endogeneity could arise due to omitted variable bias, measurement error, or reverse causality.

To mitigate these issues, we exploit lagged values of the explanatory variables as instruments. Specifically, lagged variables are assumed to be uncorrelated with the current error term while remaining correlated with the endogenous regressors. This ensures valid and strong instruments for consistent estimation.

The general form of the estimation equation is:

$$ln(Y_{imt}) = \beta_1 X_{1,imt} + \beta_2 X_{2,imt} + \ldots + \beta_k X_{k,imt} + \mu_i + \sigma_m + \lambda_t + \epsilon_{imt} \quad (2)$$

where:

- Y_{imt} represents the elective surgery average complete cases (log-transformed) for Trust i in month m in year t,
- $X_{k,it}$ denotes the kth explanatory variable, such as NHS workforce sickness rate, strike days, infrastructure investments, elective surgery incomplete cases within 50km, and the share of older adults in the Trust area,
- μ_i are Trust fixed effects to control for time-invariant unobserved heterogeneity,
- σ_i are month fixed effects to capture the seasonality
- λ_t are year fixed effects to capture time-specific shocks affecting all Trusts,
- ϵ_{it} is the error term.

The model incorporates lagged dependent and explanatory variables as both regressors and instruments, recognising the dynamic nature of incomplete case ratios and their determinants. Consistent with the monthly structure of the dataset, lagged explanatory variables refer to a one-month lag (t-1), unless otherwise specified (for some variables, such as Investments in Infrastructure the lag is one year instead). For instance, the lagged ratio of elective surgery incomplete to completed cases reflects the persistence of performance outcomes over time, while lagged explanatory variables capture delayed effects of inputs such as investments or workforce dynamics.

We employ a two-step GMM estimation procedure with robust standard errors. The first step estimates the model while the second refines weights for optimal efficiency. Standard errors are clustered at the Trust level to account for potential within-Trust correlation over time.

3 Robustness checks

Table A4 presents the GMM regression results for the log of elective surgery average completed cases at the NHS Trust level from 2018 to 2023. GMM is employed here to address potential endogeneity concerns, particularly the possibility that some explanatory variables (e.g., sickness rates or investment levels) may be correlated with unobserved shocks or measurement errors in the outcome variable. This method provides more reliable estimates compared to OLS by using internal instruments and exploiting variation over time. The results broadly corroborated the earlier OLS findings (Table 1), strengthening the robustness of the conclusions while adding greater nuance. The NHS Medical Workforce sickness rate remains a statistically significant negative predictor of data completeness across all specifications (M1-M3), with coefficients between -0.0755 and -0.0712 ($p < 0.01$). Interpreted at the 2023 mean sickness rate of 5.01, a one-unit

increase (e.g., from 5.01 to 6.01) is associated with a 7.1% to 7.5% decline in the average number of elective surgery complete cases. This translated to a roughly 0.275 to 0.288 point decrease on the 2023 log mean of 3.935, or a 6.9 to 7.3 percentage point decline relative to the average. Conversely, NHS Medical Workforce strike days did not exhibit a statistically significant relationship with data completeness in any model. Despite potential short-term disruptions, strikes appear not to materially affect overall data completeness in the longer term. The effect of regional pressures is both consistent and significant. The log of total elective surgery incomplete cases within a 50km radius is positively associated with data completeness, with coefficients ranging from 1.83 to 2.48 across models (all $p < 0.01$). At the 2023 mean of 10.533 for this variable, a 1% increase (approximately a 0.105 unit change in logs) is associated with a 0.221% to 0.284% increase in elective surgery complete cases. In practical terms, this equates to an increase of 4.7 to 6.5 percentage points relative to the 2023 mean log of average elective surgery complete cases, suggesting that competitive or spillover effects may motivate Trusts to improve performance in the presence of regional data pressures. Lagged infrastructure investment and internal elective surgery incomplete cases do not show robust statistical significance, and the share of people aged 70+ in the local area also lacks significant explanatory power in the final model. Finally, lagged elective surgery incomplete cases within Trusts is not a significant predictor, indicating that internal factors play a smaller role in this model specification. Investments in infrastructure (log) also do not exhibit a significant effect on data completeness in any of the models, consistent with the earlier findings in Table 2. The use of GMM strengthens the validity of these results by mitigating potential biases, highlighting the importance of workforce health and regional dynamics in shaping data quality while reaffirming the robustness of the earlier OLS conclusions. The consistency of key results (e.g., sickness rates, elective surgery incomplete cases in a radius of 50 KM) across methodologies strengthens their policy relevance, while the GMM-specific findings highlight the importance of accounting for spatial and dynamic dependencies when designing interventions.

The GMM estimates presented in Table A5 reinforce and expand upon the earlier findings of Table 2 for elective surgery incomplete cases over the average of elective surgery complete cases, providing additional robustness by addressing potential endogeneity concerns. The NHS medical workforce sickness rate remains a highly significant positive predictor of data incompleteness across all model specifications (M1-M3), with coefficients ranging from 0.0680 to 0.0807 ($p < 0.01$). At the 2023 mean sickness rate of 5.01, a one-unit increase is associated with a 6.8% to 8.1% rise in the log ratio of incomplete to average elective surgery complete cases, equivalent to a 0.42 to 0.48 point increase relative to the 2023 log mean of 6.125. NHS medical workforce strike activity is also significant in M1 and M2 (coefficients between 0.0449 and 0.0857, $p < 0.05$), suggesting that strike-related disruptions may contribute to higher data incompleteness; the average of 0.584 strike days in 2023 would imply an associated 5.3% rise in incomplete reporting. Strikingly, regional pressure, captured by the total elective surgery incomplete cases within 50km, is consistently associated with significant reductions in incompleteness (coefficients between -1.414 and -2.218, $p < 0.01$), indicating a potential spillover or competitive effect. A one-unit increase at the 2023 mean of 10.533 corresponds to a 15.5% to 21.2% reduction in the incomplete-to-complete case ratio. Internal dynamics show weaker but directionally consistent effects: lagged internal incompleteness is significant only in M2, and the local share of people aged 70+ does not exhibit explanatory power. Overall, these results underscore the dual importance of workforce stability and external pressures in shaping reporting performance, with GMM estimation enhancing the credibility of the findings by accounting for potential dynamic and spatial dependencies.

Finally, in Table A6, we report the GMM results, which show similar findings to those presented in Table 5.

Table A2: Skewness diagnostics for selected variables.

Variable	z = skew/SE	Pr(Skewness) p-value
Elective surgery average complete cases	1269.776	0.00
Elective surgery Incomplete over average complete cases	649.746	0.00
Total elective surgery Incomplete cases within Trusts	80.963	0.00
Total Trust HGR costs (log)	91.992	0.00
Total elective surgery Incomplete cases within 50 km	46.242	0.00
Total Trust Investments in infrastructure	1905.822	0.00

Table A3: Missing HGR costs in Trusts by NHSE region.

NHSE region name	Non-missing Trusts	Missing Trusts	Total
East of England	12	6	18
	(66.67%)	(33.33%)	(100.00%)
London	14	12	26
	(53.85%)	(46.15%)	(100.00%)
Midlands	14	18	32
	(43.75%)	(56.25%)	(100.00%)
North East and York..	13	12	25
	(52.00%)	(48.00%)	(100.00%)
North West	17	9	26
	(65.38%)	(34.62%)	(100.00%)
South East	13	10	23
	(56.52%)	(43.48%)	(100.00%)
South West	7	6	13
	(53.85%)	(46.15%)	(100.00%)
Total	90	73	163
	(55.21%)	(44.79%)	(100.00%)

Table A4: GMM Estimate effects of average elective surgery complete cases (log) at NHS Trust level, 2018-2023.

	M1	M2	M3
NHS Medical Workforce sickness rate	-0.0755***	-0.0729***	-0.0712***
	(0.0128)	(0.0144)	(0.0107)
	p=2.08e-08	p=1.05e-06	p=4.99e-10
	CI: [-0.101, -0.0502]	[-0.101, -0.0445]	[-0.0923, -0.0501]
NHS Medical Workforce days of strikes	-0.0690*	-0.0689	-0.0398*
	(0.0417)	(0.0424)	(0.0215)
	p=0.0998	p=0.106	p=0.0659
	CI: [-0.151, 0.0133]	[-0.153, 0.0147]	[-0.0822, 0.00265]
Total Trust Investments in infrastructure (log, lag)	-0.0243	-0.0245	-0.0125
	(0.0278)	(0.0279)	(0.0301)
	p=0.383	p=0.382	p=0.678
	CI: [-0.0793, 0.0306]	[-0.0796, 0.0307]	[-0.0719, 0.0469]
Total elective surgery Incomplete cases within 50km (log)	2.348***	2.482***	1.833***
	(0.408)	(0.551)	(0.512)
	p=3.83e-08	p=1.21e-05	p=0.000460
	CI: [1.543, 3.153]	[1.395, 3.570]	[0.822, 2.844]
Total elective surgery Incomplete cases within Trusts (log, lag)		-0.174	0.0217
		(0.349)	(0.285)
		p=0.619	p=0.939
		CI: [-0.862, 0.515]	[-0.541, 0.585]
Share of old people (70+) in the Trust local area			-0.246
			(1.447)
			p=0.865
			CI: [-3.106, 2.614]
Trust Fixed Effects	Yes	Yes	Yes
Year Fixed Effects	Yes	Yes	Yes
Month Fixed Effects	Yes	Yes	Yes
Observations	11,522	11,522	10,002
Hansen J statistic (p-value)	0.586	0.573	0.815

Notes: Standard errors in parentheses. p-values prefixed with 'p ='. Confidence intervals in square brackets. *** p<0.01, ** p<0.05, * p<0.1.

Table A5: GMM Estimate effects of elective surgery incomplete cases over average elective surgery complete cases (log) at NHS Trust level, 2018-2023.

	M1	M2	M3
NHS Medical sickness rate	0.0807***	0.0680***	0.0739***
	(0.0139)	(0.0132)	(0.0120)
	p=2.79e-08	p=6.42e-07	p=7.15e-09
	CI: [0.0533, 0.108]	[0.0420, 0.0940]	[0.0501, 0.0977]
NHS Medical Workforce days of strikes	0.0859*	0.0857*	0.0449**
	(0.0436)	(0.0445)	(0.0199)
	p=0.0502	p=0.0559	p=0.0256
	CI: [-8.90e-05, 0.172]	[-0.00219, 0.174]	[0.00557, 0.0843]
Total Trust Investments	0.0178	0.0164	0.00145
in infrastructure (log, lag)	(0.0265)	(0.0265)	(0.0349)
	p =0.502	p=0.538	p=0.967
	CI: [-0.0345, 0.0701]	[-0.0360, 0.0687]	[-0.0674, 0.0704]
Total elective surgery Incomplete cases	-1.622***	-2.218***	-1.414***
of Trusts within 50km (log)	(0.353)	(0.475)	(0.502)
	p = 8.32e-06	p=6.04e-06	p=0.00553
	CI: [-2.319, -0.925]	[-3.156, -1.281]	[-2.406, -0.422]
Total elective surgery Incomplete cases		0.629*	0.115
within Trust (lag)		(0.319)	(0.360)
		p = 0.0504	p=0.749
		CI: [-0.00122, 1.260]	[-0.595, 0.826]
Share of old people (70+)			1.950
in the Trust local area			(2.138)
			p = 0.363
			CI: [-2.274, 6.174]
Trust Fixed Effects	Yes	Yes	Yes
Year Fixed Effects	Yes	Yes	Yes
Month Fixed Effects	Yes	Yes	Yes
Observations	11,522	11,522	10,002
Hansen J statistic (p-value)	0.276	0.287	0.77

Notes: Standard errors in parentheses. p-values prefixed with 'p ='. Confidence intervals in square brackets. *** p<0.01, ** p<0.05, * p<0.1.

Table A6: GMM Estimate effects of average elective surgery complete cases (log) and elective surgery incomplete cases over average elective surgery complete cases (log) at NHS Trust level, Aug 2018- Feb 2023.

	**Per Capita Complete**		**Incomplete Over Average**	
NHS Medical sickness rate	-0.0726***	-0.0944***	0.0692***	0.0878***
	(0.0114)	(0.0114)	(0.0123)	(0.0121)
	p=2.04e-09	p=0	p=9.07e-08	p=0
	CI: [-0.0951, -0.0502]	[-0.117, -0.0719]	[0.0449, 0.0936]	[0.0638, 0.112]
NHS Workforce day of strikes	0.0144	-0.120**	-0.00241	0.120**
	(0.0503)	(0.0566)	(0.0509)	(0.0597)
	p=0.775	p=0.0363	p=0.962	p=0.0455
	CI: [-0.0850, 0.114]	[-0.232, -0.00775]	[-0.103, 0.0981]	[0.00247, 0.238]
Total Trust Investments in infrastructure (log, lag)	0.00724	-0.000671	-0.0211	-0.0207
	(0.0274)	(0.0378)	(0.0322)	(0.0421)
	p = 0.792	p=0.986	p=0.513	p=0.625
	CI: [-0.0469, 0.0614]	[-0.0754, 0.0741]	[-0.0846, 0.0425]	[-0.104, 0.0626]
Total elective surgery Incomplete cases of Trusts within 50km (log)	2.407***	1.702***	-2.281***	-1.753***
	(0.654)	(0.564)	(0.689)	(0.625)
	p = 0.000327	p=0.00299	p=0.00116	p=0.00568
	CI: [1.114, 3.700]	[0.588, 2.816]	[-3.642, -0.920]	[-2.987, -0.519]
Total elective surgery Incomplete cases within Trust (lag)	-0.0960	0.714**	0.678	-0.116
	(0.409)	(0.359)	(0.465)	(0.461)
	p = 0.815	p=0.0484	p=0.146	p=0.801
	CI: [-0.904, 0.712]	[0.00495, 1.423]	[-0.240, 1.596]	[-1.027, 0.794]
Share of old people (70+) in the Trust local area	0.271	-1.895	-0.314	-1.675
	(1.812)	(2.025)	(1.917)	(2.469)
	p = 0.881	p=0.351	p=0.870	p=0.498
	CI: [-3.309, 3.850]	[-5.897, 2.106]	[-4.102, 3.473]	[-6.554, 3.203]
NHS Medical Workforce Turnover (Joiners - Leavers)	-0.000129	0.00866	0.00214	0.00497
	(0.00806)	(0.0174)	(0.00985)	(0.0165)
	p = 0.987	p=0.618	p=0.828	p=0.763
	CI: [-0.0161, 0.0158]	[-0.0256, 0.0430]	[-0.0173, 0.0216]	[-0.0276, 0.0375]
NHS Admin Workforce Turnover (Joiners - Leavers)	0.0136**	0.0103	-0.0160**	-0.0215***
	(0.00582)	(0.00627)	(0.00643)	(0.00611)
	p=0.0203	p=0.103	p=0.0137	p=0.000577
	CI: [0.00215, 0.0251]	[-0.00212, 0.0227]	[-0.0288, -0.00333]	[-0.0336, -0.00943]
Total Trust HGR costs (log)		-0.134		-1.141
		(2.549)		(1.458)
		p = 0.958		p=0.435
		CI: [-5.172, 4.903]		[-4.023, 1.740]
Observations	7,608	5,291	7,608	5,291
Trust Fixed Effects	Yes	Yes	Yes	Yes
Year Fixed Effects	Yes	Yes	Yes	Yes
Month Fixed Effects	Yes	Yes	Yes	Yes
Hansen J statistic (p-value)	0.406	0.41	0.34	0.863

Notes: Standard errors in parentheses. p-values prefixed with ‘p = ‘. Confidence intervals shown as ‘CI: [lower, upper]‘. *** p<0.01, ** p<0.05, * p<0.1.

Table A7: OLS estimates of effects on average elective surgery completed cases (log) at NHS Trust level accounting for Covid (2018-2023).

	M1	M2	M3
NHS Medical Workforce sickness rate - pre-Covid	-0.0320***	-0.0352***	-0.0400***
	(0.0108)	(0.0108)	(0.00854)
	0.00306	0.00111	2.82e-06
	CI: [-0.0533, -0.0108]	[-0.0564, -0.0141]	[-0.0568, -0.0233]
NHS Medical Workforce sickness rate - post-Covid	-0.0501***	-0.0540***	-0.0517***
	(0.0143)	(0.0143)	(0.0114)
	0.000460	0.000157	6.02e-06
	CI: [-0.0781, -0.0221]	[-0.0820, -0.0260]	[-0.0741, -0.0293]
NHS Medical Workforce days of strikes	-0.0253	-0.0308	-0.000614
	(0.0312)	(0.0312)	(0.0246)
	0.418	0.323	0.980
	CI: [-0.0865, 0.0359]	[-0.0919, 0.0303]	[-0.0489, 0.0476]
Total Trust Investments	-0.00103	0.000706	0.0165**
in infrastructure (log, lag) - pre-Covid	(0.00813)	(0.00812)	(0.00758)
	0.899	0.931	0.0293
	CI: [-0.0170, 0.0149]	[0.0152, 0.0166]	[0.00166, 0.0314]
Total Trust Investments	0.00915	0.00670	0.000700
in infrastructure (log, lag) - post-Covid	(0.0113)	(0.0112)	(0.0112)
	0.416	0.551	0.950
	CI: [-0.0129, 0.0312]	[-0.0153, 0.0287]	[-0.0212, 0.0226]
Total elective surgery Incomplete cases	0.0734**	0.0675*	0.0676**
within 50km (log) - pre-Covid	(0.0359)	(0.0358)	(0.0269)
	0.0410	0.0593	0.0120
	CI: [0.00299, 0.144]	[-0.00266, 0.138]	[.0149, 0.120]
Total elective surgery Incomplete cases	0.0711**	0.0688**	0.0662**
within 50km (log) - post-Covid	(0.0352)	(0.0351)	(0.0263)
	0.0432	0.0497	0.0117
	CI: [0.00217, 0.140]	[9.65e-05, 0.138]	[0.0148, 0.118]
Total elective surgery Incomplete cases		0.189***	0.0776***
within Trust (log, lag)		(0.0238)	(0.0204)
		0	0.000143
		CI: [0.142, 0.235]	[0.0376, 0.118]
Share of old people (70+)			-0.131***
in the Trust local area			(0.0440)
			p=0.002
			CI: [-0.217, -0.0447]
Observations	11,733	11,720	10,173
R-squared	0.526	0.529	0.573
Trust Fixed Effects	Yes	Yes	Yes
Year Fixed Effects	Yes	Yes	Yes
Month Fixed Effects	Yes	Yes	Yes

Notes: Standard errors in parentheses. p-values prefixed with ‘p =’. Confidence intervals in square brackets. *** p<0.01, ** p<0.05, * p<0.1.

Table A8: OLS estimates of elective surgery incomplete over average complete cases (log) at NHS Trust level accounting for Covid (2018-2023).

	M1	M2	M3
NHS Medical Workforce sickness rate - pre-Covid	0.0410***	0.0266**	0.0319***
	(0.0114)	(0.0110)	(0.00882)
	0.000329	0.0157	0.000305
	CI: [0.0186, 0.0634]	[0.00502, 0.0483]	[0.0146, 0.0492]
NHS Medical Workforce sickness rate - post-Covid	0.0643***	0.0483***	0.0496***
	(0.0151)	(0.0146)	(0.0118)
	2.06e-05	0.000921	2.69e-05
	CI: [0.0347, 0.0938]	[0.0197, 0.0769]	[0.0264, 0.0727]
NHS Medical Workforce days of strikes	0.0492	0.0362	0.00383
	(0.0329)	(0.0318)	(0.0254)
	0.135	0.255	0.880
	CI: [-0.0154, 0.114]	[-0.0262, 0.0986]	[-0.0460, 0.0537]
Total Trust Investments	-0.0139	-0.00365	-0.0204***
in infrastructure (log, lag) - pre-Covid	(0.00857)	(0.00829)	(0.00783)
	0.105	0.660	0.00925
	CI: [-0.0307, 0.00292]	[-0.0199, 0.0126]	[-0.0358, -0.00504]
Total Trust Investments	-0.00509	-0.00891	-0.000277
in infrastructure (log, lag) - post-Covid	(0.0119)	(0.0115)	(0.0116)
	0.668	0.438	0.981
	CI: [-0.0284, 0.0182]	[-0.0314, 0.0136]	[-0.0230, 0.0224]
Total elective surgery Incomplete cases	-0.0448	-0.0667*	-0.0689**
within 50km (log) - pre-Covid	(0.0379)	(0.0365)	(0.0278)
	0.236	0.0679	0.0133
	CI: [-0.119, 0.0294]	[-0.138, 0.00491]	[-0.123, -0.0144]
Total elective surgery Incomplete cases	-0.0601	-0.0684*	-0.0672**
within 50km (log) - post-Covid	(0.0371)	(0.0358)	(0.0271)
	0.105	0.0561	0.0132
	(-0.133 - 0.0126)	(-0.139 - 0.00178)	(-0.120 - -0.0140)
Total elective surgery Incomplete cases		0.709***	0.821***
within Trust (log, lag)		(0.0242)	(0.0211)
		0	0
		CI: [0.661, 0.756]	[0.779, 0.862]
Share of old people (70+)			0.133***
in the Trust local area			(0.0454)
			p=0.0034
			CI: [0.0440, 0.222]
Observations	11,733	11,720	10,173
R-squared	0.839	0.850	0.888
Trust Fixed Effects	Yes	Yes	Yes
Year Fixed Effects	Yes	Yes	Yes
Month Fixed Effects	Yes	Yes	Yes

Notes: Standard errors in parentheses. p-values prefixed with 'p ='. Confidence intervals in square brackets. *** p<0.01, ** p<0.05, * p<0.1.

Table A9: OLS Estimate effects of average elective surgery complete cases (log) and incomplete cases over average complete cases (log) at NHS Trust level accounting for Covid, 2018-2023.

	**Per Capita complete**		**Incomplete over average complete**	
NHS Medical Workforce sickness rate pre-Covid	-0.0396***	-0.0527***	0.0307***	0.0423***
	(0.00893)	(0.00891)	(0.00921)	(0.00921)
	9.60e-06	3.48e-09	0.000842	4.40e-06
	CI: [-0.0571, -0.0220]	[-0.0702, -0.0353]	[0.0127, 0.0488	[0.0243, 0.0604]
NHS Medical Workforce sickness rate post- Covid	-0.0474***	-0.0753***	0.0469***	0.0749***
	(0.0127)	(0.0181)	(0.0130)	(0.0187)
	0.000180	3.31e-05	0.000322	6.46e-05
	CI: [-0.0722, -0.0226]	[-0.111, -0.0398]	[0.0214, 0.0725]	[0.0382, 0.112]
NHS Workforce days of strikes	-0.0493	-0.163**	0.0309	0.145*
	(0.0529)	(0.0734)	(0.0545)	(0.0758)
	0.351	0.0259	0.571	0.0566
	CI: [-0.153, 0.0543]	[-0.307, -0.0196]	[-0.0759, 0.138]	[-0.00405, 0.293]
Total Trust Investments in infrastructure (log, lag) - pre-Covid	0.0205**	0.0329***	-0.0255***	-0.0391***
	(0.00852)	(0.00961)	(0.00878)	(0.00993)
	0.0161	0.000620	0.00371	8.47e-05
	CI: [0.00381, 0.0372]	[0.0141, 0.0517]	[-0.0427, -0.00828]	[-0.0585, -0.0196]
Total Trust Investments in infrastructure (log, lag) - post -Covid	-0.00417	-0.0124	0.00217	0.0118
	(0.0141)	(0.0247)	(0.0146)	(0.0256)
	0.768	0.618	0.882	0.644
	CI: [-0.0318, 0.0235]	([-0.0608, 0.0361]	[-0.0264, 0.0307]	[-0.0383, 0.0619]
Total elective surgery Incomplete cases of Trusts within 50km (log) - pre-Covid	0.0992***	0.101***	-0.102***	-0.106***
	(0.0300)	(0.0291)	(0.0309)	(0.0300)
	0.000940	0.000498	0.000995	0.000427
	CI: [0.0404, 0.158]	[0.0443, 0.158]	[-0.162, -0.0412]	[-0.165, -0.0470]
Total elective surgery Incomplete cases of Trusts within 50km (log) - post-Covid	0.0972***	0.0944***	-0.0990***	-0.0992***
	(0.0297)	(0.0292)	(0.0306)	(0.0302)
	0.00106	0.00124	0.00122	0.00102
	CI: [0.0390, 0.155]	[0.0371, 0.152]	[-0.159, -0.0390]	[-0.158, -0.0401]
Total elective surgery Incomplete cases within Trust (log, lag)	0.0754***	0.117***	0.824***	0.776***
	(0.0238)	(0.0277)	(0.0246)	(0.0286)
	0.00157	2.42e-05	0	0
	CI: [0.0287, 0.122]	[0.0626, 0.171]	[0.776, 0.872]	[0.720, 0.832]
Share of old people (70+) in Trust local area	-0.178***	-0.263***	0.174***	0.258***
	(0.0646)	(0.0831)	(0.0666)	(0.0859)
	0.00584	0.00157	0.00904	0.00264
	CI: [-0.305, -0.0515]	[0.426, -0.100]	[0.0433, 0.304]	[0.0900, 0.427]
NHS Medical Workforce turnover - pre-Covid	-0.000499	-0.00102	0.000554	0.00171
	(0.00171)	(0.00188)	(0.00176)	(0.00194)
	0.770	0.587	0.753	0.379
	CI: [-0.00384, 0.00284]	[-0.00471, 0.00267]	[-0.00289, 0.00400]	[-0.00210, 0.00552]
NHS Medical Workforce turnover - post-Covid	-0.00845***	0.0106	0.00746***	-0.0137
	(0.00216)	(0.0224)	(0.00222)	(0.0232)
	8.98e-05	0.636	0.000792	0.555
	CI; [-0.0127, -0.00422]	[-0.0334, 0.0546]	[0.00310, 0.0118]	[-0.0591, 0.0318]
NHS Admin Workforce turnover - pre-Covid	0.00634***	0.0157***	-0.00684***	-0.0174***
	(0.000466)	(0.000742)	(0.000481)	(0.000766)
	0	0	0	0
	CI: [0.00542, 0.00725]	[0.0142, 0.0171]	[-0.00779, -0.00590]	[-0.0189, -0.0159]
NHS Admin Workforce turnover - post-Covid	0.0302***	-0.0483*	-0.0300***	0.0468*
	(0.00237)	(0.0266)	(0.00245)	(0.0275)
	0	0.0696	0	0.0890
	CI; [0.0256, 0.0349]	[-0.100, 0.00387]	[-0.0348, -0.0252]	[-0.00714, 0.101]
Total Trust HGR costs (log) - pre-Covid		-0.0770		0.125*
		(0.0729)		(0.0753)
		0.291		0.0981
		CI: [-0.220, 0.0659]		[-0.0230, 0.272]
Total Trust HGR costs (log) - post-Covid		-0.109		0.156**
		(0.0709)		(0.0733)
		0.124		0.0330
		CI: [-0.248, 0.0298]		[0.0127, 0.300]
Observations	7,773	5,504	7,773	5,504
R-squared	0.605	0.662	0.893	0.903
Trust Fixed Effects	Yes	Yes	Yes	Yes
Year Fixed Effects	Yes	Yes	Yes	Yes
Month Fixed Effects	Yes	Yes	Yes	Yes

Notes: Standard errors in parentheses. p-values prefixed with 'p ='. Confidence intervals in square brackets. *** p<0.01, ** p<0.05, * p<0.1.

Table A10: GMM Estimate effects of average elective surgery complete cases (log) at NHS Trust level accounting for Covid, 2018-2023.

	M1	M2	M3
NHS Medical sickness rate - pre-Covid	-0.0854***	-0.0820***	-0.0796***
	(0.0181)	(0.0201)	(0.0149)
	4.80e-06	6.67e-05	3.12e-07
	CI: [-0.121, -0.0497]	[-0.122, -0.0424]	[-0.109, -0.0503]
NHS Medical sickness rate - post-Covid	-0.0471**	-0.0482**	-0.0473***
	(0.0223)	(0.0219)	(0.0177)
	0.0362	0.0293	0.00835
	CI: [-0.0911, -0.00307]	[-0.0915, -0.00490]	[-0.0823, -0.0123]
NHS Workforce days of strikes	-0.0651	-0.0652	-0.0361
	(0.0417)	(0.0422)	(0.0222)
	0.120	0.125	0.106
	CI: [-0.147, 0.0172)]	[-0.149, 0.0182]	[-0.0799, 0.00780]
Total Trust Investments	0.0131	0.0113	0.0202
in infrastructure (log, lag) - pre-Covid	(0.0566)	(0.0577)	(0.0592)
	0.817	0.845	0.734
	CI: [-0.0986, 0.125]	[-0.103, 0.125]	[-0.0968, 0.137]
Total Trust Investments	-0.0951	-0.0921	-0.0807
in infrastructure (log, lag) - post-Covid	(0.124)	(0.127)	(0.110)
	0.444	0.468	0.466
	CI: [-0.340, 0.150]	[-0.342, 0.158]	[-0.299, 0.137]
Total elective surgery Incomplete cases	2.398***	2.493***	1.875***
of Trusts within 50km (log)	(0.455)	(0.598)	(0.511)
	4.10e-07	4.85e-05	0.000341
	CI: [1.499, 3.297]	[1.312, 3.674]	[0.865, 2.886]
Total elective surgery Incomplete cases	2.393***	2.484***	1.858***
of Trusts within 50km (log)	(0.427)	(0.571)	(0.491)
	8.02e-08	2.32e-05	0.000221
	CI: [1.550, 3.235]	[1.357, 3.612]	[0.888, 2.828]
Total elective surgery Incomplete cases		-0.122	0.0507
within Trust (log, lag)		(0.357)	(0.290)
		0.733	0.861
		CI; [-0.827, 0.583]	[-0.523, 0.624]
Share of old people (70+)			-0.137
in the Trust local area			(1.091)
			p = 0.900
			CI: [-2.293, 2.018]
Observations	11,522	11,522	10,002
Hansen J statistic (p-value)	0.226	0.218	0.749
Trust Fixed Effects	Yes	Yes	Yes
Year Fixed Effects	Yes	Yes	Yes
Month Fixed Effects	Yes	Yes	Yes

Notes: Standard errors in parentheses. p-values prefixed with ‘p =’. Confidence intervals in square brackets. *** p<0.01, ** p<0.05, * p<0.1.

Table A11: GMM Estimate effects of elective surgery incomplete cases over average elective surgery complete cases (log) at NHS Trust level accounting for Covid, 2018-2023.

	M1	M2	M3
NHS Medical sickness rate - pre-Covid	0.0957***	0.0828***	0.0844***
	(0.0204)	(0.0202)	(0.0186)
	5.59e-06	6.61e-05	1.20e-05
	CI: [0.0554, 0.136]	[0.0428, 0.123]	[0.0476, 0.121]
NHS Medical sickness rate - post-Covid	0.0397*	0.0408*	0.0390**
	(0.0222)	(0.0219)	(0.0196)
	0.0765	0.0636	0.0479
	CI: [-0.00426, 0.0836]	[-0.00234, 0.0840]	[0.000359, 0.0776]
NHS Workforce days of strikes	0.0804*	0.0372*	
	(0.0433)	(0.0436)	(0.0215)
	0.0694	0.0673	0.0864
	CI; [-0.00635, 0.165]	[-0.00577, 0.166]	[-0.00538, - 0.0797]
Total Trust Investments	-0.0495	-0.0344	-0.0876*
in infrastructure (log, lag) - pre-Covid	(0.0342)	(0.0356)	(0.0465)
	0.149	0.336	0.0614
	CI; [-0.117, 0.0179]	[-0.105, 0.0360]	[-0.179, 0.00424]
Total Trust Investments	0.144**	0.112*	0.183*
in infrastructure (log, lag) - post-Covid	(0.0690)	(0.0662)	(0.0941)
	0.0387	0.0910	0.0541
	CI: [0.00752, 0.280]	[-0.0181, 0.243]	[0.00328, 0.369]
Total elective surgery Incomplete	-1.770***	-2.175***	-1.526***
of Trusts within 50km (log)	(0.385)	(0.500)	(0.481)
	8.29e-06	2.31e-05	0.00186
	CI: [-2.530, -1.010]	[-3.162, -1.188]	[-2.477, -0.574]
Total elective surgery Incomplete	-1.861***	-2.243***	-1.589***
of Trusts within 50km (log)	(0.392)	(0.486)	(0.499)
	4.24e-06	7.51e-06	0.00175
	CI: [-2.634, -1.088]	[-3.201, -1.284]	[-2.575, -0.604]
Total elective surgery Incomplete cases		0.460	0.0313
within Trust (log, lag)		(0.357)	(0.406)
		0.199	0.939
		[-0.245, 1.165]	[-0.770, 0.833]
Share of old people (70+)			-0.182
in the Trust local area			(2.482)
			p = 0.942
			CI: [-5.087, 4.723]
Observations	11,522	11,522	10,002
Hansen J statistic (p-value)	0.250	0.250	0.708
Trust Fixed Effects	Yes	Yes	Yes
Year Fixed Effects	Yes	Yes	Yes
Month Fixed Effects	Yes	Yes	Yes

Notes: Standard errors in parentheses. p-values prefixed with 'p ='. Confidence intervals in square brackets. *** p<0.01, ** p<0.05, * p<0.1.

Table A12: GMM Estimate effects of average elective surgery complete cases (log) and elective surgery incomplete cases over average elective surgery complete cases (log) at NHS Trust level accounting for Covid, Aug 2018- Feb 2023.

	**Per Capita Complete**		**Incomplete Over Average**	
NHS Medical sickness rate pre-Covid	-0.0803***	-0.109***	0.0730***	0.0950***
	(0.0165)	(0.0154)	(0.0175)	(0.0154)
	2.80e-06	6.26e-11	5.24e-05	5.78e-09
	CI: [-0.113, -0.0477]	[-0.139, -0.0782	[0.0384, 0.108]	[0.0647, 0.125]
NHS Medical sickness rate post-Covid	-0.0452*	-0.0402	0.0367	0.0402
	(0.0265)	(0.0262)	(0.0274)	(0.0260)
	0.0899	0.126	0.183	0.124
	CI: [-0.0974, - 0.00712	[-0.0919, - 0.0115]	[-0.0174, 0.0907]	[-0.0111, 0.0915]
NHS Workforce day of strikes	-0.00508	-0.184***	0.0206	0.167***
	(0.0658)	(0.0573)	(0.0684)	(0.0630)
	0.939	0.00162	0.763	0.00906
	CI: [-0.135, 0.125]	[-0.297, -0.0708]	[-0.115, 0.156]	[0.0421, 0.291]
Total Trust Investments	0.0429	0.00298	-0.117**	-0.0218
in infrastructure (log, lag) - pre-Covid	(0.0541)	(0.0384)	(0.0459)	(0.0445)
	0.429	0.938	0.0115	0.624
	CI: [-0.0640, 0.150]	[-0.0730, 0.0789]	[-0.208, -0.0267]	[-0.110, 0.0661]
Total Trust Investments	-0.143	-0.115	0.334**	0.0472
in infrastructure (log, lag) - post-Covid	(0.112)	(0.289)	(0.143)	(0.339)
	0.204	0.690	0.0213	0.889
	CI: [-0.364, 0.0784]	CI: [-0.686, 0.455]	CI: [0.0503, 0.617]	CI: [-0.622, 0.717]
Total elective surgery Incomplete cases	2.403***	1.699***	-2.299***	-1.684***
of Trusts within 50km (log) - pre-Covid	(0.634)	(0.557)	(0.692)	(0.630)
	0.000216	0.00272	0.00113	0.00833
	CI: [1.151, 3.655]	[0.598, 2.800]	[-3.667, -0.930]	[-2.929, -0.440]
Total elective surgery Incomplete cases	2.384***	1.586***	-2.378***	-1.683***
of Trusts within 50km (log) - post-Covid	(0.614)	(0.529)	(0.713)	(0.610)
	0.000154	0.00317	0.00108	0.00653
	CI: [1.171, 3.597]	[0.541, 2.630]	[-3.788, -0.968]	[-2.888, -0.478]
Total elective surgery Incomplete cases	-0.0490	0.769**	0.491	-0.187
within Trust (log, lag)	(0.398)	(0.379)	(0.498)	(0.481)
	0.902	0.0440	0.326	0.698
	CI;[-0.835, 0.737]	[0.0209, 1.518]	[-0.493, 1.475]	[-1.137, 0.763]
Share of old people (70+)	0.317	-3.894*	-3.022	-1.803
	(1.246)	(2.132)	(2.945)	(3.546)
	0.800	0.0698	0.307	0.612
	CI: [-2.144, 2.778]	[-8.106, 0.319]	[-8.842, 2.798]	[-8.810, 5.204]
NHS Medical Workforce Turnover - pre-Covid	0.0234	0.00754	-0.0161	0.00483
	(0.0156)	(0.0179)	(0.0199)	(0.0177)
	0.136	0.674	0.422	0.785
	CI: [-0.00744, 0.0543]	[-0.0278, 0.0429]	[-0.0554, 0.0233]	[-0.0302, 0.0399]
NHS Medical Workforce Turnover - post-Covid	-0.0180**	-0.0132	0.0290**	0.0303
	(0.00724)	(0.116)	(0.0124)	(0.101)
	0.0138	0.910	0.0209	0.765
	CI: [-0.0323, -0.00373]	[-0.243, 0.217]	[0.00445, 0.0535]	[-0.170, 0.230]
NHS Admin Workforce Turnover - pre-Covid	0.00902	0.0118*	-0.0110*	-0.0217***
	(0.00606)	(0.00630)	(0.00650)	(0.00634)
	0.138	0.0635	0.0938	0.000780
	CI: [-0.00295, 0.0210]	[-0.000672, 0.0242]	[-0.0238, 0.00188]	[-0.0343, -0.00922]
NHS Admin Workforce Turnover - post-Covid	0.0384***	-0.132	-0.0462***	0.0338
	(0.0120)	(0.116)	(0.0123)	(0.0943)
	0.00170	0.256	0.000255	0.721
	CI: [0.0146, 0.0621]	[-0.362, 0.0969]	[-0.0705, -0.0218]	[-0.153, 0.220]
Total Trust HGR costs (log)- pre-Covid		-0.781		-0.918
		(2.509)		(1.685)
		0.756		0.587
		CI: [-5.740, 4.177]		[-4.249, 2.412]
Total Trust HGR costs (log) - post-Covid		-1.918		-1.061
		(2.675)		(1.385)
		0.474		0.445
		CI: [-7.204, 3.367]		CI: [-3.798, 1.676]
Observations	7,608	5,291	7,608	5,291
Hansen J statistic (p-value)	0.251	0.248	0.244	0.301
Trust Fixed Effects	Yes	Yes	Yes	Yes
Year Fixed Effects	Yes	Yes	Yes	Yes
Month Fixed Effects	Yes	Yes	Yes	Yes

Notes: Standard errors in parentheses. p-values prefixed with ‘p = ‘. Confidence intervals shown as ‘CI: [lower, upper]‘. *** p<0.01, ** p<0.05, * p<0.1.
